# Biomimetic Bone Marrow Monocyte Membrane‐Fused Extracellular Vesicles for Targeted Therapy of Myocardial Infarction

**DOI:** 10.1002/advs.75445

**Published:** 2026-04-27

**Authors:** Jiaxin Song, Hao Yang, Qiqi Zhang, Wenqin Zhou, Rui Wang, Yuxing Xie, Emeli Chatterjee, Guoping Li, Jizong Jiang, Qiulian Zhou, Cuimei Zhao

**Affiliations:** ^1^ Institute of Geriatrics (Shanghai University) Affiliated Nantong Hospital of Shanghai University (The Sixth People's Hospital of Nantong) and School of Life Science Shanghai University Nantong China; ^2^ Cardiac Regeneration and Ageing Lab Institute of Cardiovascular Sciences Shanghai Engineering Research Center of Organ Repair School of Life Science Joint International Research Laboratory of Biomaterials and Biotechnology in Organ Repair (Ministry of Education) Shanghai University Shanghai China; ^3^ Cardiovascular Division of the Massachusetts General Hospital and Harvard Medical School Boston Massachusetts USA; ^4^ Department of Anesthesia Critical Care and Pain Medicine Massachusetts General Hospital and Harvard Medical School Boston Massachusetts USA; ^5^ Department of Cardiology Shanghai Tongji Hospital Tongji University School of Medicine Shanghai China

**Keywords:** delivery system, extracellular vesicle, myocardial infarction, mononuclear cell membrane, targeted therapy

## Abstract

Myocardial infarction (MI) represents a major public health challenge. Extracellular vesicles (EVs) hold considerable promise as therapeutics for cardiovascular disorders. However, the targeted delivery of them to the heart has received relatively limited research. Acute MI is often accompanied by severe inflammation. After MI, numbers of monocytes/macrophages are rapidly mobilized from the circulation and accumulate within the ischemic myocardial tissue. This recruitment is driven by the inflammatory homing signals emanating from the injured cardiac region. In this work, we develop a biomimetic nanovesicle by fusing membranes isolated from bone marrow mononuclear cells (Mon) with extracellular vesicles derived from healthy human plasma (M‐hEV). This biomimetic delivery platform achieves site‐specific accumulation at damaged vascular endothelial cells and cardiomyocytes by leveraging two key molecular recognition mechanisms: the monocyte chemoattractant protein‐1 (MCP‐1)/C‐C chemokine receptor 2 (CCR2) and intercellular adhesion molecule‐1 (ICAM‐1)/CD11b axes. The results indicate that M‐hEV can inhibit apoptosis of vascular endothelial cells and cardiomyocytes, promote angiogenesis and regulate macrophage polarization at the cellular and animal levels. Furthermore, M‐hEV can home to the MI heart, reduce the infarct size, reduce the inflammation level, and improve cardiac function. This biomimetic system provides a novel approach to explore new targeted drugs for MI treatment.

## Graphical abstract

This study develops a biomimetic delivery system (M‐hEV) by fusing monocyte membranes with extracellular vesicles for targeted therapy of damaged cardiac tissue. The system homes to injured myocardium through specific molecular pathways. In a myocardial infarction model, M‐hEV effectively accumulates in the heart, reduces infarct size, alleviates inflammation, and significantly enhances cardiac function, demonstrating a promising therapeutic strategy.

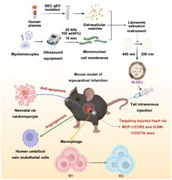

## 1 Introduction

The incidence and mortality of acute myocardial infarction (MI) are increasing, presenting a serious threat to human health [1]. Traditional drugs have a single therapeutic target and are unable to reverse established myocardial injury [2]. The heart's unique environment, characterized by high blood flow velocity and an endothelial barrier, poses significant challenges to drug accumulation and retention at the target site. This limitation has hindered the development of precision therapeutics specifically designed for MI. While novel treatments based on stem cells, genes, and nano‐biomedical materials have emerged, their clinical application is largely limited due to in situ administration requirements [3, 4, 5]. Owing to the multifaceted pathophysiology of cardiovascular disorders, there is a pressing demand for novel therapeutic approaches and next‐generation pharmacological agents.

Extracellular vesicles (EVs) have demonstrated multifaceted therapeutic potential in cardiovascular disease management, spanning diagnosis, therapeutic intervention, and prognostic assessment, owing to their distinctive biophysical and biochemical properties [6]. A large amount of evidence has shown that the contents (including miRNAs and heat shock protein family) carried by different types of cell‐derived EVs collectively contribute to multimodal cardiac repair [7, 8]. Our previous studies found that EVs from healthy human plasma (hEV) rich in miRNA‐486, can repair the damaged myocardium [9]. Circulating blood‐derived EVs offer advantages over cell‐derived EVs in terms of availability, yield, and effectiveness, making them a viable alternative. However, their accumulation in the heart is limited, and few studies have explored targeted delivery via systemic circulation. EVs administered through the circulatory system are often cleared by the mononuclear phagocyte system, thereby reducing their targeting specificity and therapeutic efficacy [10]. Genetic engineering strategies, including overexpression of specific genes in parent cells or direct EV modification, coupled with surface conjugation of cardiac‐targeting peptides (e.g., APWHLSSQYSRT or CSTSMLKAC), can enhance EV targeting specificity and therapeutic efficacy [11, 12]. However, the mechanism of action of these peptides targeted by phage display is still unclear and needs to be further verified.

Monocytes/macrophages are key mediators of inflammatory responses following MI. Particularly during the initial phase, macrophages, lymphocytes and mast cells are first recruited and infiltrated into the infarcted myocardium, passing through the necrotic muscle fibers and gradually engulf the infarcted myocardium [13, 14]. Studies have shown that integrins, cell adhesion molecules and chemokines are the main molecules involved in monocyte migration and adhesion. Monocytes express chemokine receptors, such as CXC‐type chemokine receptor 2 (CXCR2), C‐C motif chemokine receptor 1 (CCR1), CCR2, and CCR5, which interact with specific chemokine ligands, including monocyte chemoattractant protein‐1 (MCP‐1), Chemokine (C‐C motif) ligand 7 (CCL7), CCL3 (macrophage inflammatory protein‐1α, MIP‐1α), and CCL5, to facilitate integrin‐mediated monocyte adhesion to the vascular endothelium and cross the endothelial barrier into cardiac tissue [15]. Other adhesion molecules, for example, macrophage receptor 1 (Mac1), platelet endothelial cell adhesion molecule (PECAM1), α4β1, p‐selectin glycoprotein ligand 1 (PSGL1), and lymphocyte function associated antigen 1 (LFA1) have also been reported to participate in leukocyte cell migration and adhesion [16]. The post‐infarction heart has a recruitment effect on monocytes, and this homing effect can be an important target for precision interventions in cardiovascular diseases. Nevertheless, the mechanism of targeting cardiac injury at the cellular and organ levels remains to be further elucidated.

In this study, a bone‐marine‐derived mononuclear cell membrane (Mon)‐fused hEV delivery system was constructed for targeted therapy of MI. We found that monocycle‐fused hEV (M‐hEV) was biaxial homing to the infarcted heart of mice via MCP‐1/CCR2 and intercellular adhesion molecule‐1 (ICAM‐1)/CD11b, thereby promoting internalization by oxygen‐glucose deprivation and reperfusion (OGD/R)‐injured human umbilical vein endothelial cells (HUVECs) and neonatal rat cardiomyocytes (NRCMs). After inhibition of CCR2 and CD11b, this targeting was significantly weakened. The M‐hEV biomimetic delivery system enhanced the targeting of the infarcted heart and repairs the damaged myocardium by regulating the phenotype of macrophages, promoting neovascularization, and inhibiting cardiomyocyte apoptosis. Accordingly, it exhibited multifaceted therapeutic functions in the targeted intervention of MI, offering a novel conceptual framework for developing MI‐targeted therapeutics.

## 2 Results

### 2.1 Characterization of Hybrid Nanovesicles

**FIGURE 1:**
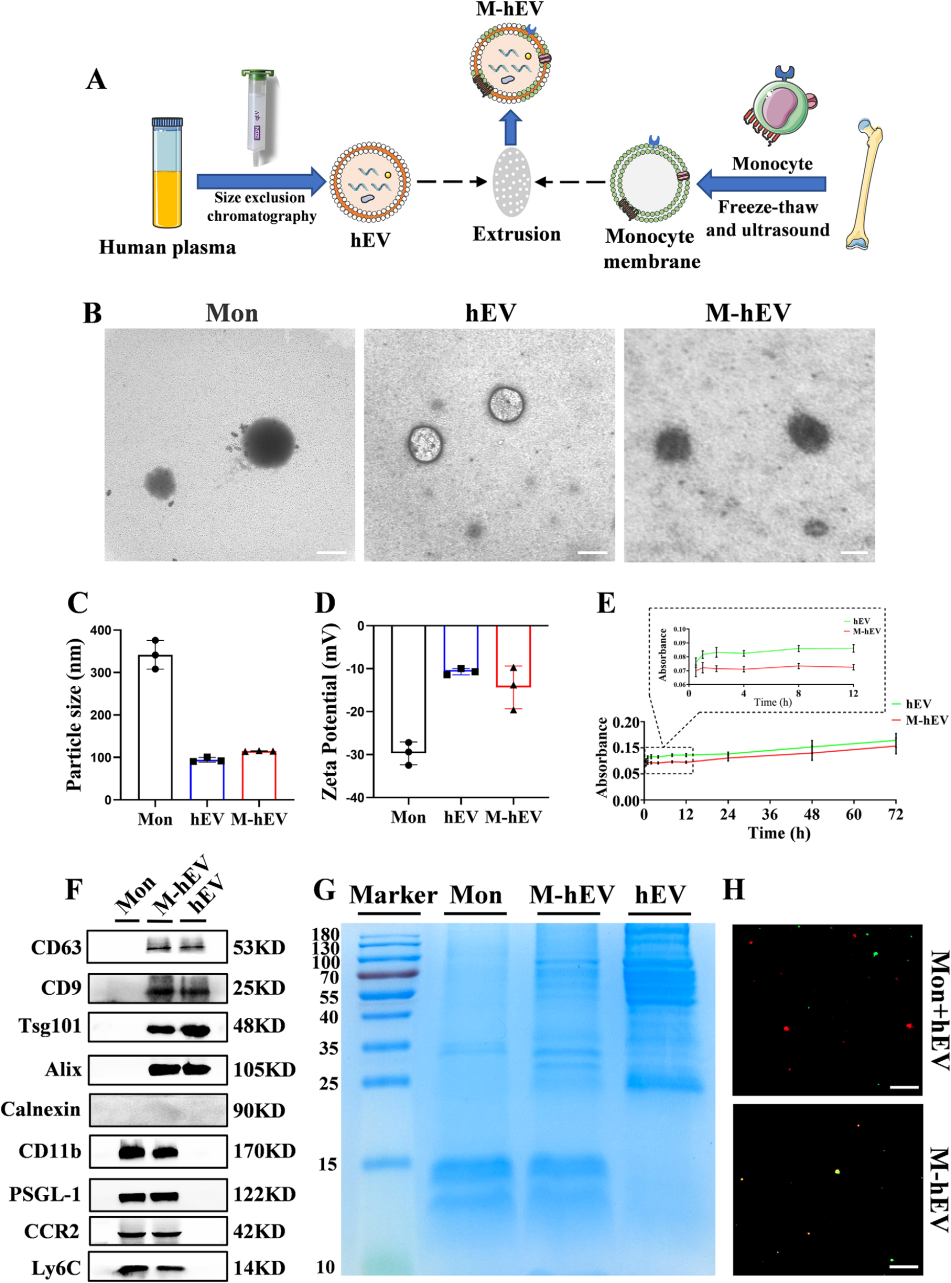
Isolation, purification, and membrane fusion verification of M‐hEV. (A) Schematic diagram of isolation of Mon, hEV and preparation of M‐hEV. (B) The morphology of Mon, hEV, and M‐hEV was characterized by TEM (n = 3) (Scale bar = 100 nm). (C) The particle sizes of Mon, hEV, and M‐hEV were determined by NTA (n = 3). (D) Zetasizer Nano ZSE was employed to characterize the surface charge properties of Mon, hEV, and M‐hEV (n = 3). (E) UV–vis spectrophotometric assessment (absorbance at 590 nm) of hEV and M‐hEV stability in 10% FBS at predetermined time points (n = 6). (F) WB analysis for CD63, CD9,Tsg101, Alix, Calnexin, CD11b, PSGL‐1, CCR2, and Ly6C in Mon, hEV, and M‐hEV (n = 3). (G) Protein profiling of Mon, hEV, and M‐hEV via coomassie brilliant blue staining (n = 3). (H) Laser scanning confocal microscopy was used to assess the fusion between DiD‐labeled Mon and DiO‐labeled hEV. The fusion preparation was performed either through a straightforward mixing approach or via extrusion(n = 3) (Scale bar = 50 µm).

Flow cytometric immunophenotyping was conducted to delineate the cellular heterogeneity and immunological identity of the isolated bone marrow mononuclear cell fraction. The results showed that Ly6C^+^CCR2^+^ monocytes accounted for 84.74% ± 1.77% of the population, consistent with previous findings (Figure S1) [17]. Monocyte‐derived nanovesicles and hEV were hybridized via membrane extrusion (Figure 1A). The initial morphological assessment of Mon nanovesicles, hEV, and M‐hEV was characterized using transmission electron microscopy (TEM). The Mon nanovesicles displayed a predominantly spherical morphology, while the hEV showed a typical extracellular vesicle structure (Figure 1B). The M‐hEV, resulting from cell membrane fusion, displayed a nanoscale morphology, indicating successful integration of the Mon nanovesicles with hEV. As shown in Figure 1C, nanoparticle tracking analysis (NTA) revealed mean hydrodynamic diameters of Mon, hEV, and M‐hEV were 341.63 ± 27.68 nm, 94.43 ± 4.60 nm, and 114.90 ± 0.61 nm, respectively. The ‌electrokinetic potentials of Mon, hEV, and M‐hEV were –29.70 ± 2.17 mV, –10.68 ± 0.58 mV, and –14.36 ± 4.06 mV, respectively (Figure 1D). The particle sizes and the absolute value of the Zeta potentials of M‐hEV were slightly larger than those of hEV, which might be attributed to the modification of Mon. Figure 1E demonstrated that both hEV and M‐hEV maintained favorable colloidal stability in a 10% fetal bovine serum (FBS) of PBS solution under ambient temperature conditions for 72 h, suggesting minimal aggregation or sedimentation under these conditions. Figure 1F presented the specific protein expressions of Mon, hEV, and M‐hEV, as determined by western blot (WB) analysis. CD11b, PSGL‐1, CCR2, and Ly6C were specific markers of monocytes while hEV specifically expressed CD63, CD9, Tsg101, and Alix, and their specific protein expressions were distinct. CD47 was expressed on both Mon and M‐hEV, but absent in native hEV, indicating a potential mechanism for improved systemic circulation persistence (Figure S2A). Furthermore, M‐hEV encompassed all the above‐mentioned specific protein expressions of Mon and hEV. The coomassie brilliant blue staining results also revealed that M‐hEV had total protein expression of Mon and hEV (Figure 1G). It suggested that M‐hEV retained relatively complete biological structures and active substances of Mon and hEV after extrusion fusion. To further validate the membrane fusion of Mon and hEV, Mon and hEV were stained with DiD and DiO fluorescent dyes, respectively. Figure 1H indicated that the physically co‐incubation of Mon and hEV failed to induce efficient membrane fusion, whereas extrusion‐mediated processing resulted in substantial colocalization of red fluorescently labeled Mon and green fluorescently labeled DiO‐labeled hEV, as evidenced by overlapping fluorescence signals, suggesting that the two membranes were successfully fused. Furthermore, both total protein and RNA content were effectively retained in extruded M‐hEV and native hEV, indicating that this preparation method did not destroy the native extracellular vesicle structure (Figure S2B,C). The nano‐flow cytometry analysis revealed that the proportion of CD63^+^/CCR2^+^ M‐hEV in the hybrid vesicles was 65.08% ± 5.00% (Figure S2D). These results demonstrate that our M‐hEV fusion protocol is highly efficient, achieving a formation yield exceeding 60%, thereby significantly improving subsequent target specificity and therapeutic efficacy. Furthermore, immunoelectron microscopy images demonstrated that CD11b and CCR2 were predominantly distributed at the outer rim of the hybrid vesicles (Figure S2E). This indicated that M‐hEV predominantly exhibited a right‐side‐out membrane orientation, which aligned with prior experimental observations reported in the previous study [18].

### 2.2 M‐hEV Enhanced HUVEC and NRCM Entry and Inhibited Their Apoptosis

**FIGURE 2:**
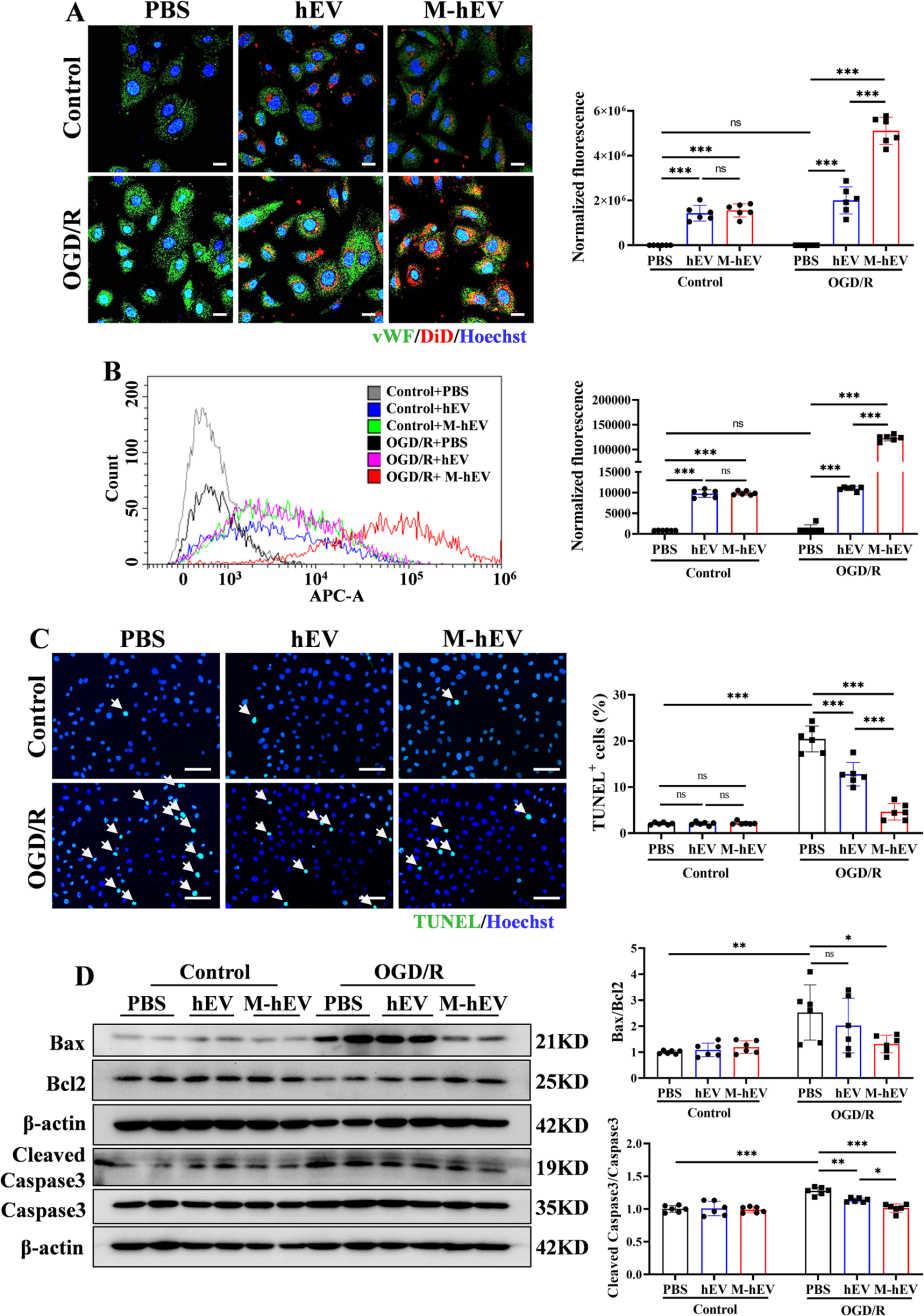
Cellular uptake and apoptosis of HUVECs under both normoxic conditions and OGD/R stress after treatment with hEV and M‐hEV. (A) Laser confocal microscopy visualization of PBS, hEV, and M‐hEV internalization in vWF‐labeled HUVECs, followed by semi‐quantitative analysis of fluorescence signal intensity (n = 6) (Scale bar = 20 µm). (B) Flow cytometry plots and statistics of fluorescence intensities of PBS, hEV, and M‐hEV in HUVECs using flow cytometry (n = 6). (C) TUNEL assay images depicting number of apoptotic cells, along with quantitative analysis of the proportion of TUNEL‐positive cells (n = 6) (Scale bar = 100 µm). (D) WB analysis for apoptosis‐related proteins in HUVECs, with quantification normalized to the Control + PBS group (n = 6). The data were presented as the mean ± standard deviation. Two‐way ANOVA with Bonferroni correction were performed (A‐D), ns, not significant, * *p* < 0.05, ** *p* < 0.01, *** *p* < 0.001.

To investigate M‐hEV cellular uptake, hEV and M‐hEV were both labeled with DiD. In addition, vWF‐labeled HUVECs and α‐actinin‐labeled NRCMs were subjected to OGD/R. Confocal microscopy and semi‐quantitative analysis (Figure 2A) revealed no significant differences in fluorescence intensities between hEV and M‐hEV in non‐OGD/R‐induced HUVECs. However, under OGD/R stress, M‐hEV exhibited enhanced uptake by injured HUVECs compared to hEV (*p* < 0.001). Similar results were obtained through quantitative analysis of flow cytometry. Under OGD/R stress, compared with hEV, more M‐hEV were internalized in HUVECs (123358.40 ± 5056.06 vs 10971.33 ± 437.68, *p* < 0.001) (Figure 2B). The increased uptake of M‐hEV by injured HUVECs suggested a therapeutic benefit, potentially mediated by apoptosis inhibition. To validate this, M‐hEV's anti‐apoptotic efficacy was assessed using TUNEL and WB assays. As shown in Figure 2C, TUNEL staining indicated that OGD/R could induce HUVEC apoptosis. Both hEV and M‐hEV were capable of inhibiting OGD/R‐induced HUVEC apoptosis. Compared with the hEV group, the M‐hEV group could more effectively protect vascular endothelial cells (4.63% ± 1.62% vs 12.78% ± 2.32%, *p* < 0.001), suggesting that M‐hEV could better protect HUVECs from OGD/R damage. WB analysis revealed that M‐hEV exhibited a significantly stronger inhibitory effect than hEV on both Bax/Bcl2 ratio and the cleaved Caspase3/total Caspase3 ratio in HUVECs (*p* < 0.05; Figure 2D).

**FIGURE 3:**
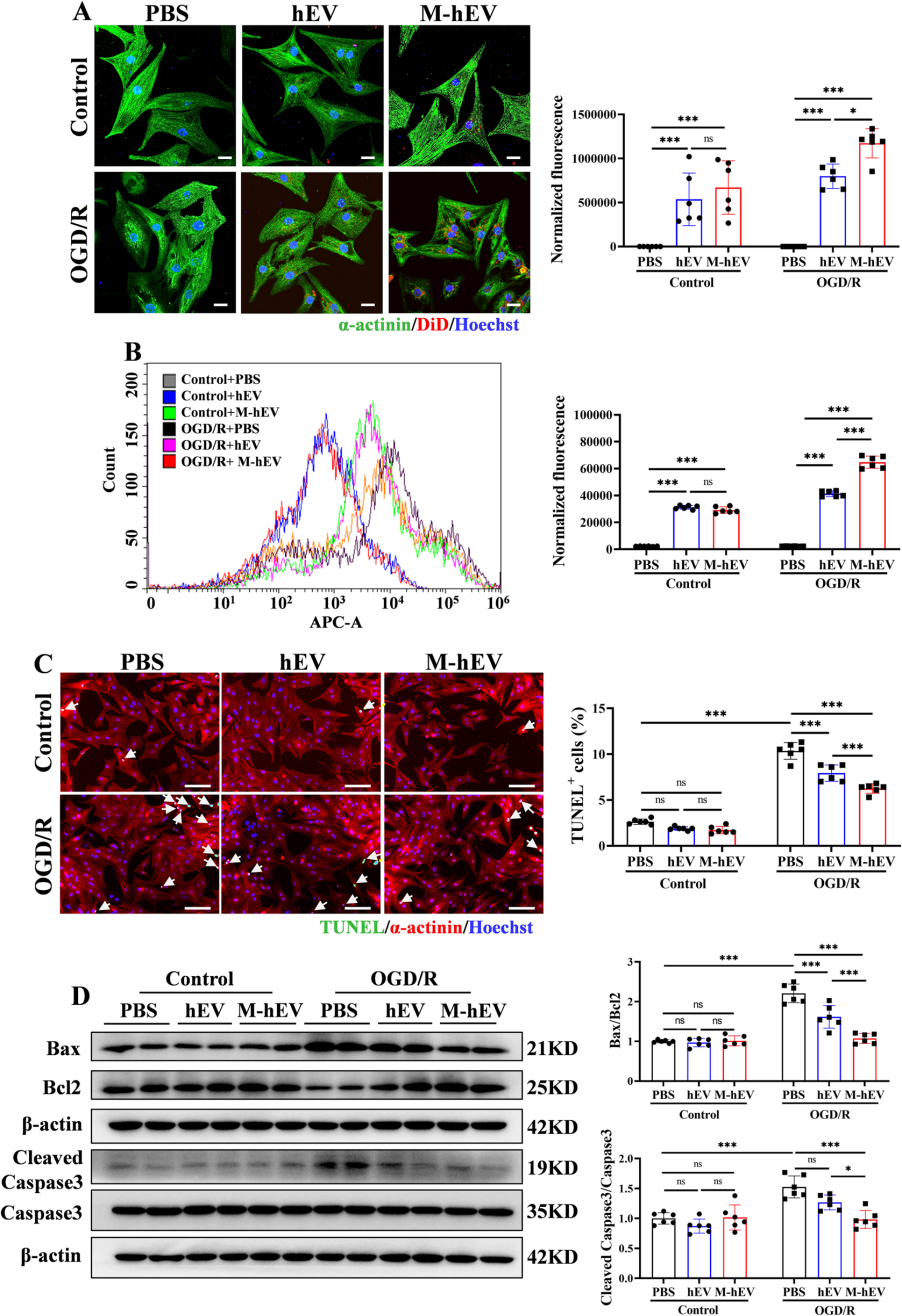
Cellular uptake and apoptosis of NRCMs under both normoxic conditions and OGD/R stress after treatment with M‐hEV. (A) Laser confocal microscopy visualization of PBS, hEV, and M‐hEV internalization in α‐actinin–immunolabeled NRCMs, followed by semi‐quantitative analysis of fluorescence signal intensity (n = 6) (Scale bar = 20 µm). (B) Flow cytometric quantification of fluorescence intensity and statistical assessment for PBS, hEV, and M‐hEV in NRCMs (n = 6). (C) TUNEL assay images depicting apoptotic NRCM, along with quantitative analysis of the proportion of TUNEL‐positive cells (n = 6) (Scale bar = 100 µm). (D) Western blot analysis for apoptosis‐related proteins in NRCMs, with quantification normalized to the Control + PBS group (n = 6). The data were presented as the mean ± standard deviation. Two‐way ANOVA with Bonferroni correction was performed (A‐D), ns, not significant, * *p* < 0.05, *** *p* < 0.001.

We subsequently validated the cellular internalization and protective effects of M‐hEV in a cardiomyocyte model. As shown in Figure 3A, confocal laser and semi‐quantitative results indicated that in non‐OGD/R‐induced NRCMs, the cellular uptake of hEV and M‐hEV by NRCMs showed no statistical differences. Under OGD/R conditions, M‐hEV exhibited enhanced cellular uptake in NRCMs than hEV (*p* < 0.05). Similar results were obtained through quantitative analysis of flow cytometry. M‐hEV treatment resulted in markedly enhanced fluorescence intensity relative to hEV treatment (64802.18 ± 4085.35 vs 41336.17 ± 1910.16, *p* < 0.001) (Figure 3B). As depicted in Figure 3C, TUNEL staining revealed that OGD/R induced NRCM apoptosis. Compared with the hEV group, the M‐hEV group could more effectively protect NRCMs against OGD/R injury (6.25% ± 0.47% vs 7.93% ± 0.81%, *p* < 0.001). M‐hEV exhibited a significantly stronger inhibitory effect than hEV on expression of apoptosis‐related proteins in NRCMs subjected to OGD/R injury, as confirmed by WB analysis (*p* < 0.05; Figure 3D). Furthermore, to identify the specific microRNAs (miRNAs) in hEV that mediate anti‐apoptotic effects, we used reverse transcription quantitative PCR (RT‐qPCR) to quantify the abundance of several candidate miRNAs in EVs (Figure S3A–E). It was found miR‐21‐5p was the sole species significantly upregulated in both hEV (*p* < 0.001) and M‐hEV (*p* < 0.001) relative to EVs obtained from mesenchymal stem cells (MSC‐EVs) (Figure S3B). Notably, miR‐21‐5p abundance did not differ significantly between hEV and M‐hEV, suggesting that this miRNA was constitutively enriched and functionally involved in the anti‐apoptotic activity of hEV, with its expression level preserved following membrane fusion modification. These findings implied that M‐hEV were more efficient in entering injured HUVECs and NRCMs, thereby achieving a better therapeutic outcome.

### 2.3 M‐hEV Targeted Injured HUVECs and NRCMs Through I‐CAM‐1/CD11b and MCP‐1/CCR2 Axes

**FIGURE 4:**
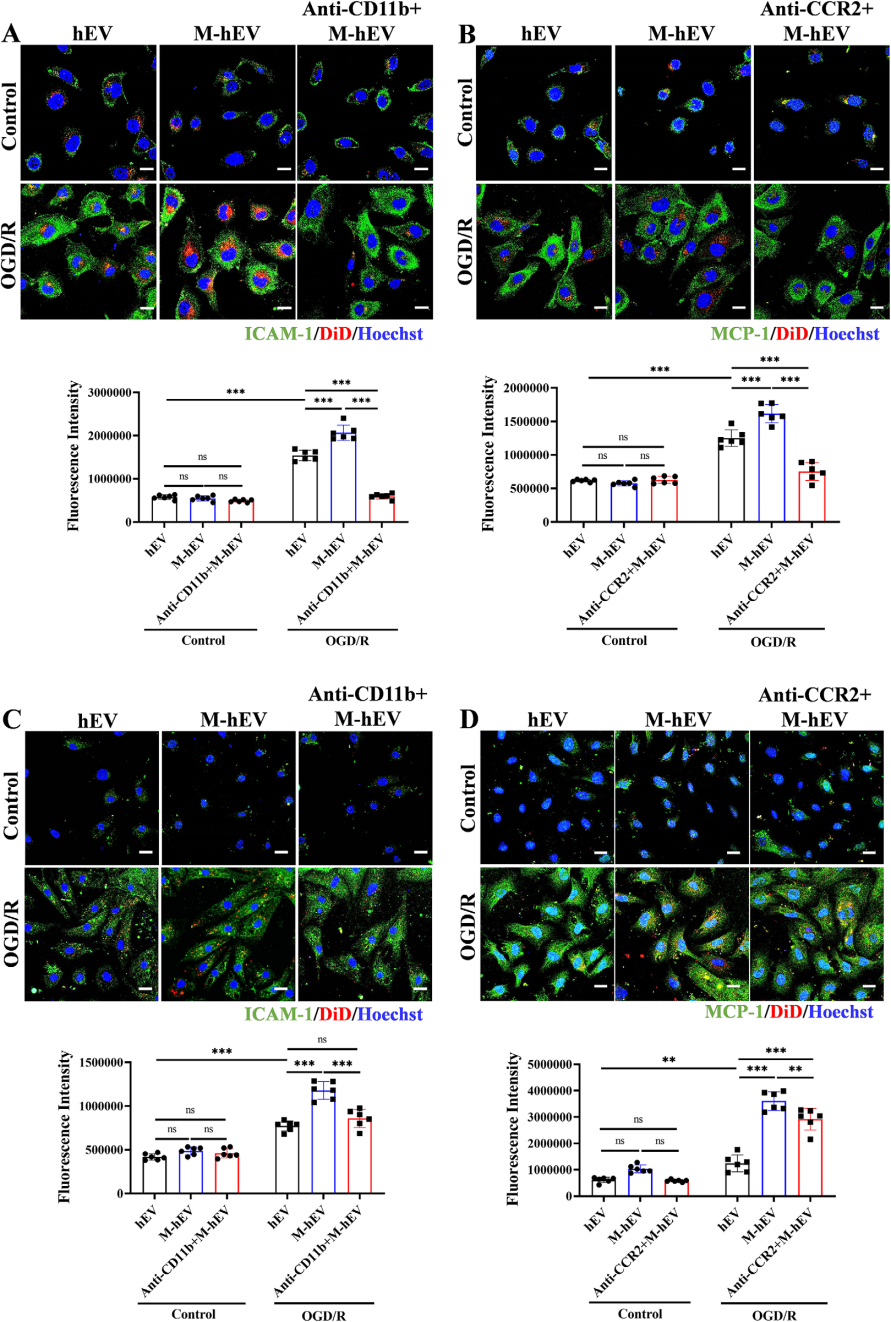
Cellular uptake of DiD‐labeled M‐hEV in HUVECs and NRCMs with anti‐antibody blocking under both normoxic conditions and OGD/R stress. (A) Imaging and fluorescence intensity statistical analysis was conducted using a confocal laser microscope on DiD‐labeled M‐hEV in ICAM‐1‐labeled HUVECs with or without anti‐CD11b blocking (n = 6) (Scale bar = 20 µm). (B) Imaging and fluorescence intensity statistical analysis was conducted using a confocal laser microscope on DiD‐labeled M‐hEV in MCP‐1‐labeled HUVECs with or without anti‐CCR2 blocking (n = 6) (Scale bar = 20 µm). (C) Imaging and fluorescence intensity statistical analysis was conducted using a confocal laser microscope on DiD‐labeled M‐hEV in ICAM‐1‐labeled NRCMs with or without anti‐CD11b blocking (n = 6) (Scale bar = 20 µm). (D) Imaging and fluorescence intensity statistical analysis was conducted using a confocal laser microscope on DiD‐labeled M‐hEV in MCP‐1‐labeled NRCMs with or without anti‐CCR2 blocking (n = 6) (Scale bar = 20 µm). The data were presented as the mean ± standard deviation. Two‐way ANOVA with Bonferroni correction was performed (A‐D), ns, not significant, ** *p* < 0.01, *** *p* < 0.001.

Monocyte homing is mediated by a coordinated interplay of integrins, adhesion molecules, and chemokine receptors [19]. To elucidate M‐hEV targeting, OGD/R‐induced HUVECs and NRCMs were used. CD11b and CCR2 on M‐hEV were blocked with antibodies, then incubated with cells. Confocal microscopy images revealed that OGD/R‐induced HUVECs overexpressed ICAM‐1 and MCP‐1, providing binding sites for M‐hEV's CD11b and CCR2 as shown in Figure 4A,B. Semi‐quantitative analysis also indicated that the fluorescence intensities of treatment with anti‐CD11b or anti‐CCR2 antibodies markedly reduced the cellular uptake of DiD‐labeled M‐hEV vs. M‐hEV (both *p* < 0.001). Similarly, OGD/R stress markedly enhanced ICAM‐1 and MCP‐1 expression levels in NRCMs. As shown in Figure 4C,D, the fluorescence intensities of DiD‐labeled M‐hEV were significantly decreased compared with the M‐hEV group after blocking by anti‐CD11b (*p* < 0.001) and anti‐CCR2 antibody (*p* < 0.01). Our findings demonstrated that OGD/R‐induced injury upregulated ICAM‐1 and MCP‐1 expression in both HUVECs and NRCMs. Meanwhile, M‐hEV possessed a homing property of the mononuclear cell membrane, which was associated with its expression of CD11b and CCR2. Inhibition of CD11b and CCR2 in M‐hEV significantly decreased their entry OGD/R‐induced HUVECs and NRCMs, suggesting that M‐hEV might play a targeting role through the ICAM‐1/CD11b and MCP‐1/CCR2 axes.

### 2.4 Pro‐Angiogenic Capacity and Polarization of Macrophages of M‐hEV In Vitro

**FIGURE 5:**
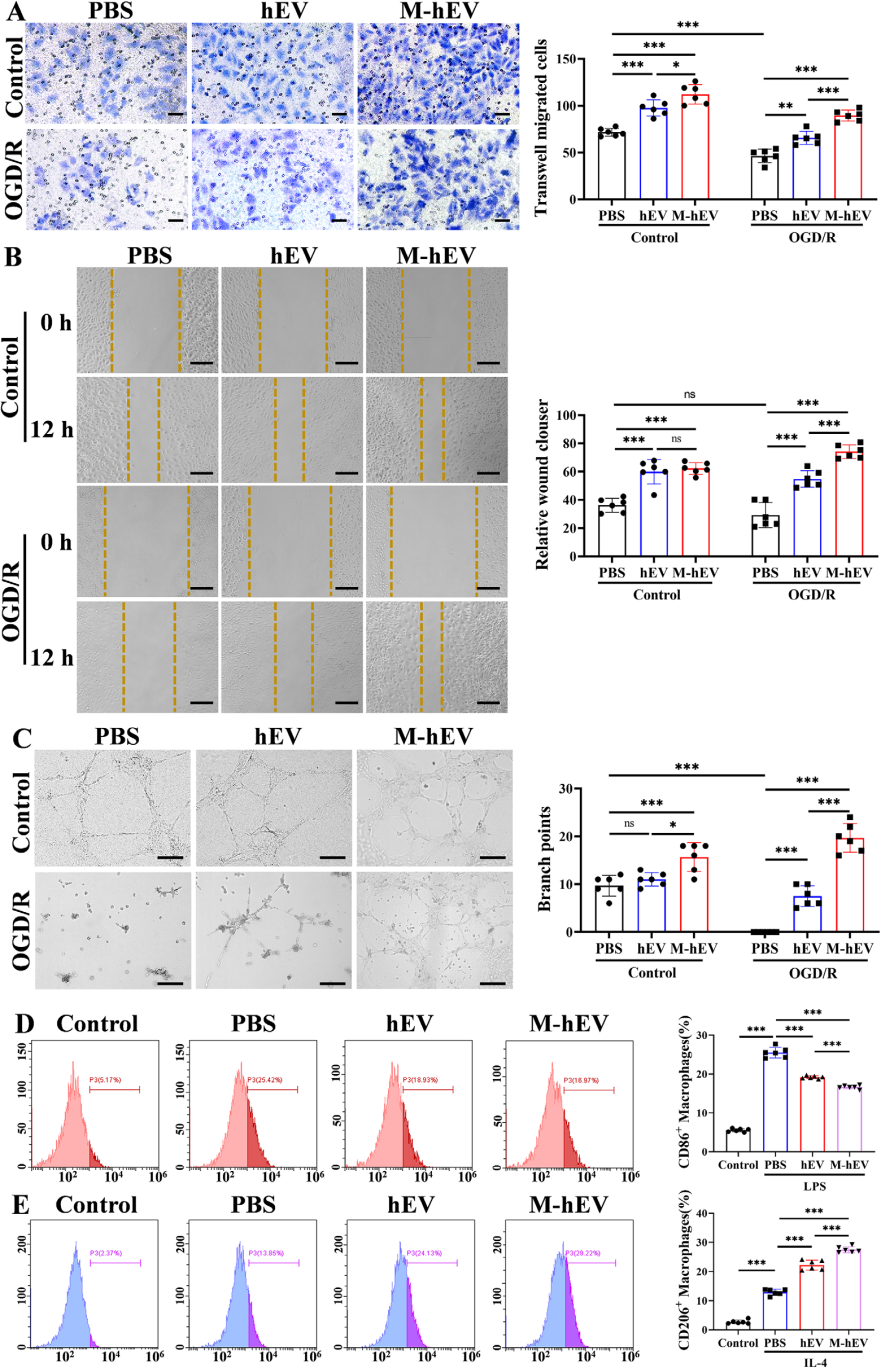
The regulatory impacts of M‐hEV on the migration, vascular lumen formation of HUVECs and polarization of macrophages in vitro both normoxic conditions and OGD/R stress. (A) Crystal violet staining and quantitative analysis for HUVECs in transwell model following treatment with PBS, hEV, and M‐hEV (n = 6) (Scale bar = 50 µm). (B) Cell migration assay and quantitative evaluation of HUVEC migratory capacity following administration of PBS, hEV, and M‐hEV (n = 6) (Scale bar = 100 µm). (C) Images depicting vascular lumen formation by HUVECs, along with quantitative assessment of branch point numbers following administration of PBS, hEV, and M‐hEV (n = 6) (Scale bar = 100 µm). (D) Representative flow cytometric dot plots depicting M1‐polarized macrophages (CD86^+^) and quantitative analysis of the percentage of CD86^+^ cells within the total macrophage population (n = 6). (E) Representative flow cytometric dot plots depicting M2‐polarized macrophages (CD206^+^) and quantitative analysis of the percentage of CD206^+^ cells within the total macrophage population (n = 6). The data were presented as the mean ± standard deviation. Two‐way ANOVA (A‐C), one‐way ANOVA (D‐E) with Bonferroni correction were performed, ns, not significant, * *p* < 0.05, ** *p* < 0.01, *** *p* < 0.001.

The pro‐angiogenic capacity of M‐hEV was examined by evaluating influence on HUVEC motility and in vitro angiogenic capacity, as assessed by capillary‐like network formation. Transwell migration and wound healing assays showed hEV and M‐hEV promoted HUVEC migration, with or without OGD/R stress. M‐hEV exhibited greater efficacy, significantly enhancing HUVEC migration compared to hEV (*p* < 0.001, Figure 5A,B). The tube formation assay indicated that OGD/R stimulation reduced the lumen formation ability of HUVECs. Both hEV and M‐hEV increased the total branching length in HUVECs compared to those treated with PBS alone. HUVECs exposed to M‐hEV exhibited a significantly higher total number of branch points than those treated with native hEV under OGD/R stress (*p* < 0.001, Figure 5C), suggesting that M‐hEV could enhance the proangiogenic effects of hEV on vascular endothelial cells, which was beneficial for repairing injured vessels.

Our previous studies have demonstrated that hEV alleviated inflammation induced by myocardial ischemia‐reperfusion injury (I/RI) through regulating macrophage subtypes [20]. Herein, the RAW264.7 cell model was employed to explore the effect of hEV and M‐hEV on macrophage polarization after induction by lipopolysaccharide (LPS) and interleukin‐4 (IL‐4). The results of flow cytometry indicated that hEV and M‐hEV markedly reduced M1 macrophage counts while concurrently elevating the proportion of M2 macrophages (Figure 5D,E). Among them, M‐hEV significantly promoted the polarization of macrophages toward the M2 phenotype vs. hEV (*p* < 0.001). It implied that M‐hEV might improve the immune microenvironment after MI by regulating the macrophage subtype.

### 2.5 M‐hEV Targeted MI Heart Through ICAM‐1/CD11b and MCP‐1/CCR2 Axes

**FIGURE 6:**
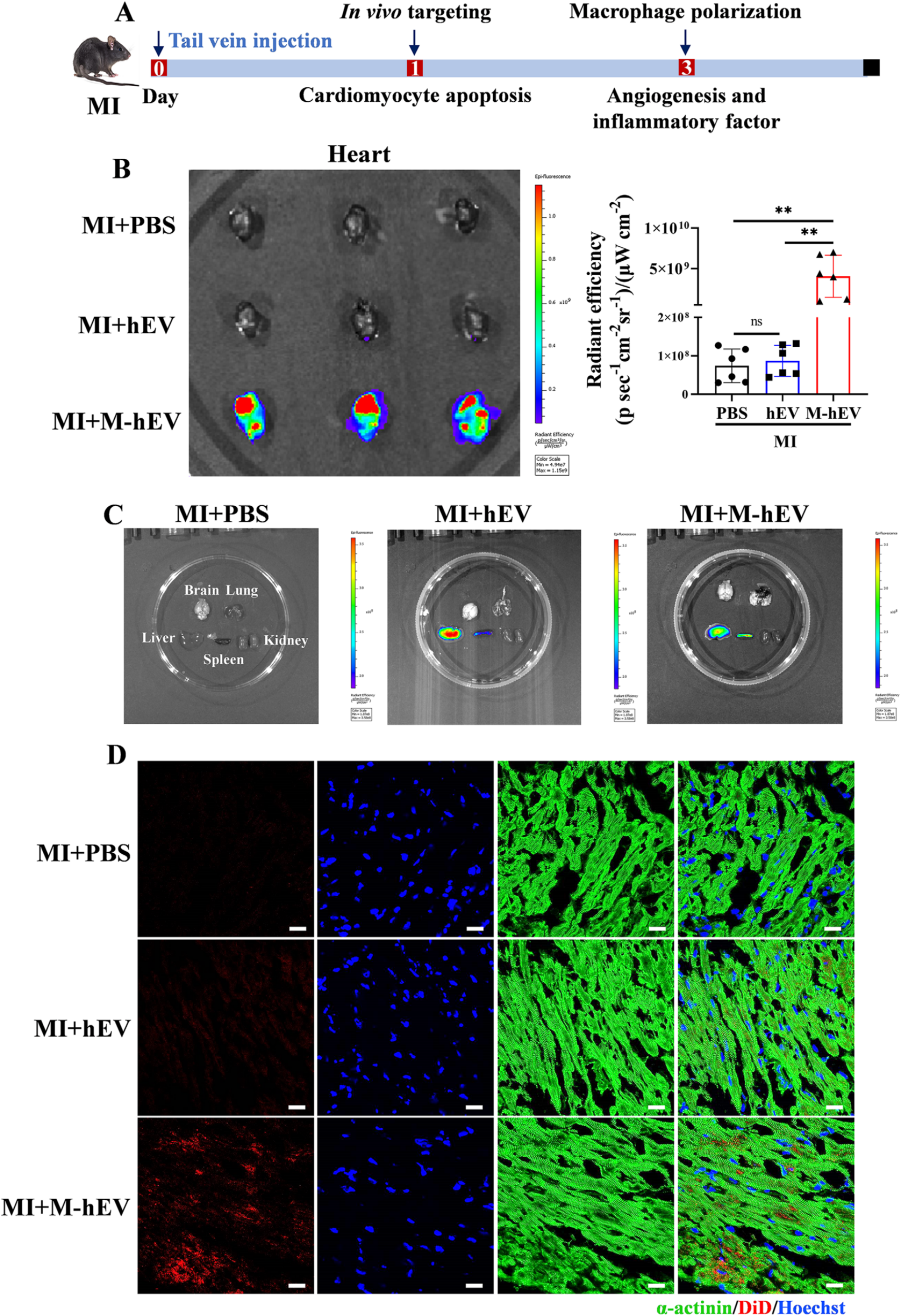
The in vivo targeting of M‐hEV. (A) Schematic diagram of the administration route and detection protocol for MI mice. (B) Semi‐quantitative assessment of cardiac fluorescence signal intensity (n = 6). (C) Fluorescence imaging of *ex vivo* main organs, including brains, lungs, livers, spleens, and kidneys from MI mice after being treated with DiD‐labeled M‐hEV for 24 h (n = 6). (D) Colocalization of DiD‐labeled M‐hEV with cardiomyocytes in the infarcted heart was examined by laser confocal microscopy. Nuclear DNA was visualized using Hoechst 33342, while cardiomyocyte sarcomeres were immunolabeled with α‐actinin (n = 6) (Scale bar = 20 µm). The data were presented as the mean ± standard deviation. One‐way ANOVA with Bonferroni correction was performed (B), ns, not significant, ** *p* < 0.01.

The detection protocol of M‐hEV for the treatment of acute MI (24 h and 3 days) was shown in Figure 6A. To investigate the cardiac homing capability of M‐hEV following MI, MI‐induced mice received tail‐vein injections of PBS, DiD‐labeled hEV, or DiD‐labeled M‐hEV. 24 h post‐injection, a markedly enhanced DiD fluorescence signal was detected in the myocardial infarct zone of the M‐hEV group via a small animal in vivo imaging system (Figure 6B). Quantitative assessment of fluorescence signals confirmed a statistically significant increase in myocardial accumulation of M‐hEV compared with hEV (*p* < 0.01). Native hEV exhibited minimal cardiac retention via systemic circulation, with no significant difference in heart fluorescence intensity vs. PBS‐treated controls. The biodistribution analysis revealed that M‐hEV predominantly localized to the liver and spleen, while showing negligible accumulation in the brains, lungs, and kidneys, mirroring nanoparticle biodistribution trends (Figure 6C). The fluorescence intensities of these isolated organs also verified these conclusions (Figure S4A–E). M‐hEV localization in MI hearts was assessed via immunofluorescence staining of heart sections. M‐hEV predominantly accumulated in the infarct area, internalized by cardiomyocytes (Figure 6D), likely facilitated by regional cytokine secretion and upregulated protein expression on cardiomyocytes.

To confirm M‐hEV's targeting mechanism, CD11b and CCR2 were blocked with antibodies, resulting in significantly reduced M‐hEV accumulation in infarcted myocardium, as demonstrated by *ex vivo* fluorescence imaging (Figure S5A). This suggested that CCR2 and CD11b mediated M‐hEV homing to injured myocardium. M‐hEV accumulated preferentially in infarcted myocardium, targeting both damaged cardiomyocytes and activated vascular endothelium through the ICAM‐1/CD11b and MCP‐1/CCR2 signaling axes, thereby enhancing site‐specific therapeutic delivery (Figure S5B). This dual‐targeting mechanism contributed to the therapeutic efficacy of M‐hEV in MI hearts.

### 2.6 M‐hEV Exerted Cardioprotective Effects Via Apoptosis Inhibition, Angiogenesis Promotion, and Immune Microenvironment Modulation In Vivo

**FIGURE 7:**
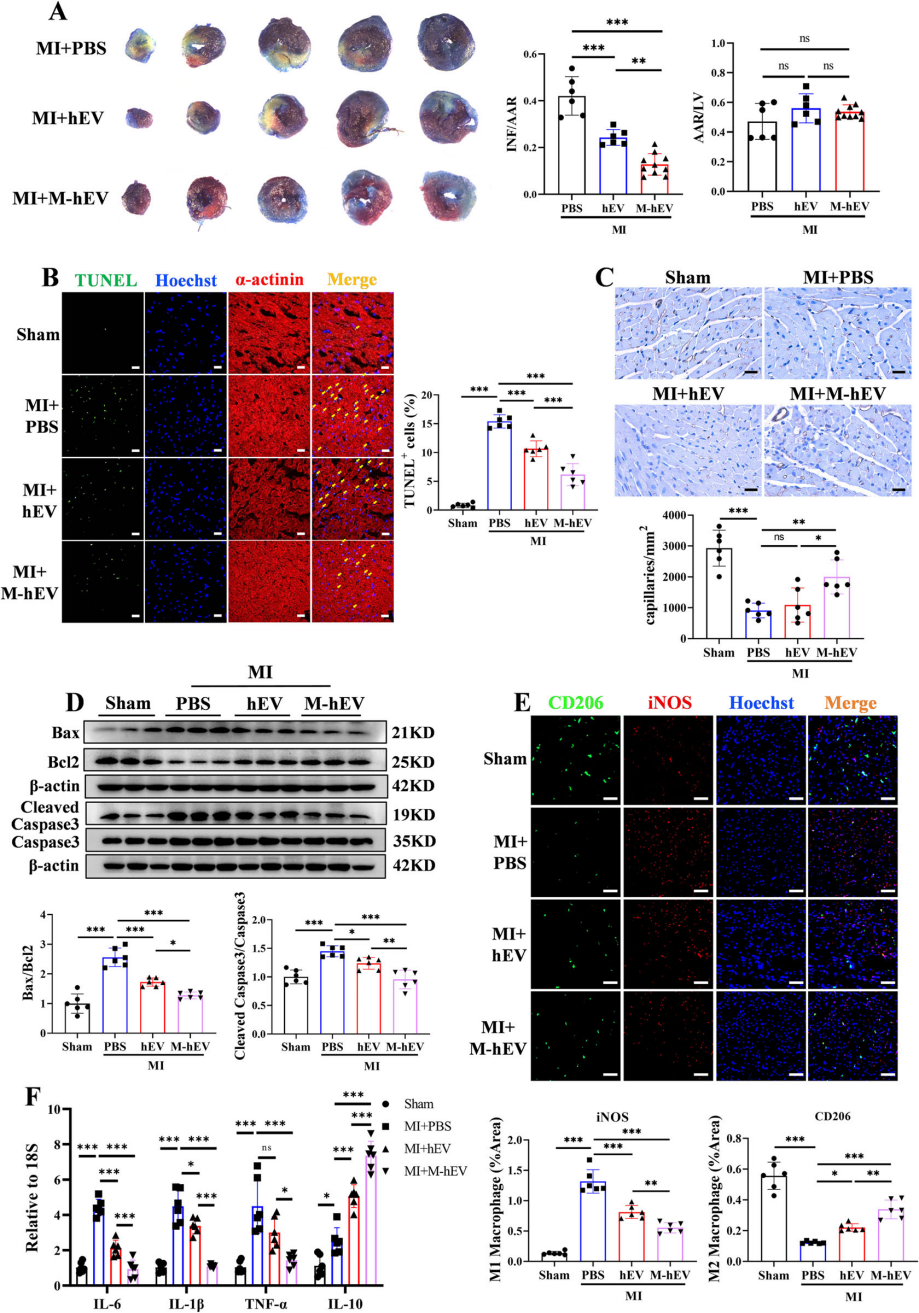
Therapeutic effects of M‐hEV in murine model of acute MI. (A) Infarct size and myocardial area‐at‐risk were quantified using triphenyl tetrazolium chloride (TTC) staining, enabling calculation of both the INF/AAR ratio and the AAR/LV ratio in PBS (n = 6), hEV (n = 6), and M‐hEV (n = 10)‐treated MI mice for 24 h. (B) TUNEL combined with α‐actinin immunofluorescence staining in cardiac tissue sections from MI mice (n = 6) (Scale bar = 20 µm). (C) Immunohistochemistry was used to investigate the expression of CD31 and quantitatively analyze the number of new capillaries in infarcted hearts (n = 6) (Scale bar = 50 µm). (D) WB analysis for apoptosis‐related proteins in myocardial tissue from MI‐induced mice, with quantification normalized to the Sham group (n = 6). (E) Immunofluorescence staining was used to identify and quantify iNOS^+^ and CD206^+^ macrophage subpopulations in the infarcted heart (n = 6) (Scale bar = 50 µm). (F) The mRNA expression of *IL‐6*, *IL‐1β*, *TNF‐α*, and *IL‐10* were quantified in cardiac tissue using RT‐qPCR, with quantification normalized to the Sham group (n = 6). The data were presented as the mean ± standard deviation. One‐way ANOVA with Bonferroni correction was performed (A‐E), ns, not significant, * *p* < 0.05, ** *p* < 0.01, *** *p* < 0.01.

To assess M‐hEV's therapeutic efficacy in MI, MI mice were administrated hEV or M‐hEV. The triphenyl tetrazolium chloride (TTC) staining demonstrated a significant decrease in myocardial infarct area in hEV (*p* < 0.001) and M‐hEV (*p* < 0.001) vs. PBS after 24 h of treatment (Figure 7A). M‐hEV showed lower infarct‐to‐area‐at‐risk (INF/AAR) ratio vs. hEV (*p* < 0.01). The area‐at‐risk‐to‐left‐ventricular‐weight ratio (AAR/LV) showed no statistically significant differences across experimental groups, indicating comparable myocardial injury extent across experimental conditions. TUNEL assay revealed that hEV and M‐hEV reduced cardiomyocyte apoptosis vs. PBS, with M‐hEV exhibiting greater anti‐apoptotic effects (6.15% ± 1.74% vs. 10.65% ± 1.24% TUNEL^+^ cells, *p* < 0.001) (Figure 7B). Cardiac tissue angiogenesis was assessed 3 days post‐hEV/M‐hEV administration. Immunohistochemistry revealed increased capillary formation in M‐hEV‐treated hearts vs. hEV (*p* < 0.05), indicating M‐hEV enhanced neovascularization and myocardial repair (Figure 7C). Additionally, M‐hEV effectively lowered the Bax/Bcl2 protein ratio (*p* < 0.05) and the cleaved Caspase3/Caspase3 ratio (*p* < 0.01) in infarcted myocardium compared with hEV (Figure 7D). Additionally, immunofluorescence results indicated that MI led to an increase in inducible nitric oxide synthase–positive (iNOS^+^) M1 macrophages (*p* < 0.001) and a decrease in CD206^+^ M2 macrophages (*p* < 0.001) vs. sham group. M‐hEV exhibited a stronger reversal effect compared with the hEV group, markedly reducing the proportion of M1 macrophages (*p* < 0.01) and concurrently increasing that of M2 macrophages (*p* < 0.01) (Figure 7E). Additionally, M‐hEV treatment for 3 days significantly enhanced the cardiac immune response. Compared with the hEV group, M‐hEV reduced the mRNA levels of pro‐inflammatory cytokine genes, including *IL‐6* (*p* < 0.001), *IL‐1β* (*p* < 0.001), and *tumor necrosis factor‐α* (*TNF‐α*) (*p* < 0.05), while elevating the anti‐inflammatory cytokine *IL‐10* (*p* < 0.001), thereby better alleviating inflammation after MI (Figure 7F). These results suggested that M‐hEV could significantly reduce the infarct area, inhibit cardiomyocyte apoptosis, promote neovascularization, and regulate the immune microenvironment, thereby further enhancing the treatment of MI.

### 2.7 M‐hEV Attenuated Adverse Cardiac Remodeling Following MI

**FIGURE 8:**
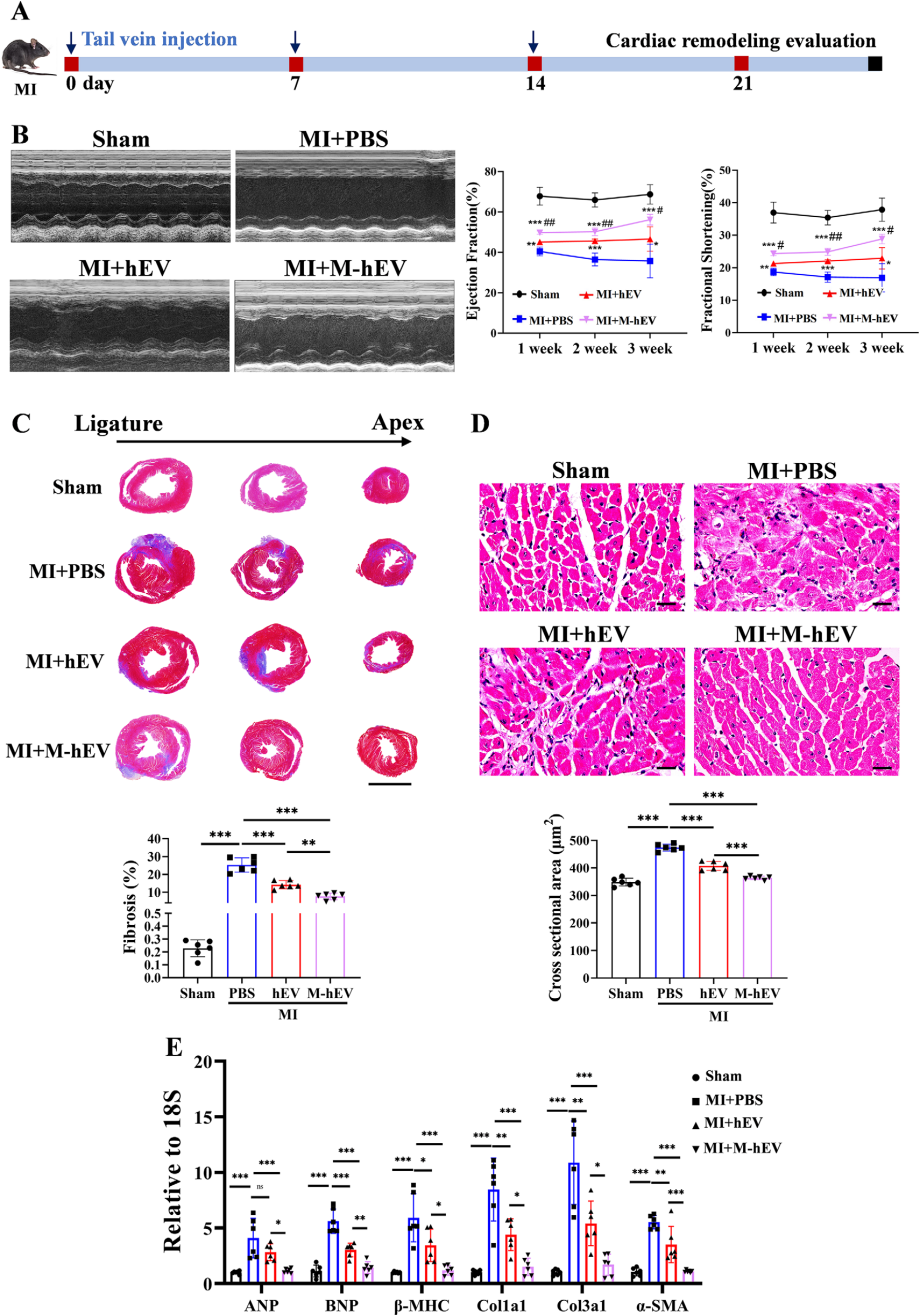
M‐hEV protected the heart from MI after 3 weeks of treatment. (A) Schematic illustration of drug administration frequency in MI mice. (B) Left ventricle EF and FS were measured by echocardiography every week (Sham: n = 6, PBS: n = 7, hEV: n = 8, M‐hEV: n = 6). (C) Cardiac fibrosis severity was assessed through Masson staining applied to multiple myocardial tissue sections (n = 6) (Scale bar = 5 mm). (D) The cardiac histomorphology and structural abnormalities was evaluated by H&E staining (n = 6) (Scale bar = 50 µm). (E) RT‐qPCR was performed to measure mRNA levels of cardiac hypertrophy and fibrosis markers, including *ANP*, *BNP*, *β‐MHC*, *Col1a1*, *Col3a1*, and *α‐SMA* in myocardial tissue samples, with quantification normalized to the Sham group (n = 6). The data were presented as the mean ± standard deviation. One‐way ANOVA with Bonferroni correction was performed (B‐E), ns, not significant, * *p* < 0.05, ** *p* < 0.01, *** *p* < 0.001.

Figure 8A presented the therapeutic administration regimen of M‐hEV on cardiac function post‐MI. Cardiac function was assessed via echocardiography three weeks after M‐hEV administration, with left ventricular ejection fraction (EF) and fractional shortening (FS) serving as the primary functional endpoints. Compared with hEV, M‐hEV showed a significantly improved EF (56.18% ± 2.39% vs 46.61% ± 5.67%, *p* < 0.05) and FS (28.83% ± 1.34% vs 22.90% ± 3.07%, *p* < 0.05) (Figure 8B). Our data indicated that M‐hEV enhanced cardiac function in all groups during the first, second, and third weeks of treatment. In addition, M‐hEV treatment significantly reduced fibrosis vs. hEV in Masson's staining, indicating improved cardiac remodeling (7.65% ± 1.70% vs 14.23% ± 2.07%, *p* < 0.01; Figure 8C). Hematoxylin‐eosin (H&E) staining at 3 weeks post‐MI showed increased myocardial area in MI mice vs. sham controls. M‐hEV treatment reduced myocardial area vs. hEV (*p* < 0.001, Figure 8D). RT‐qPCR analysis further revealed M‐hEV downregulated genes involved in pathological remodeling and fibrosis, including *ANP* (*p* < 0.05), *BNP* (*p* < 0.01), *β‐MHC* (*p* < 0.05), *Col1a1* (*p* < 0.05), *Col3a1* (*p* < 0.05), and *α‐SMA* (*p* < 0.001) vs. hEV (Figure 8E). Consequently, our study demonstrated that M‐hEV effectively attenuated cardiac dysfunction at three weeks post‐MI.

### 2.8 Biocompatibility of M‐hEV

M‐hEV cytotoxicity was preliminarily evaluated by CCK‐8 assay. Data analysis indicated that hEV and M‐hEV had negligible cytotoxic effects on HUVECs (Figure S6A) and NRCMs (Figure S6B) at 48 h. In vivo, H&E staining was carried out on additional major organs of the mouse, including the brains, livers, spleens, lungs, and kidneys 24 h post‐treatment. The results revealed no tissue damage with hEV or M‐hEV (Figure S6C). These findings indicated that systemic delivery of M‐hEV exhibited a favorable safety profile comparable to that of hEV and PBS, supporting its further evaluation.

## 3 Discussion

The heart's unique anatomy and high hemodynamic flow pose significant challenges for targeted drug delivery, hindering drug accumulation and retention. Exogenous EVs due to their size effects comparable to those of synthetic nanoparticles, are rapidly cleared by the host immune system, comprising monocytes and tissue macrophages, leading to off‐target distribution in liver, kidney, spleen, and other organs, or swift elimination [21]. Cardiac‐targeting peptide‐based EV modification techniques have been developed for cardiovascular disease treatment. Examples included CSTSMLKAC‐modified cardiac spheroid‐derived cell‐EVs and APWHLSSQYSRT‐Lamp2b‐EVs co‐loaded with Cur and miR‐144‐3p, enabling targeted delivery [11, 22]. However, the mechanism through which these peptides target the heart has not been further clarified. Recent advances in cell membrane biomimetic techniques have enabled EV modification. Platelet membrane‐coated EVs retained platelet‐derived adhesive components and targeting ability to damaged endothelium. In I/RI murine models, these engineered EVs selectively accumulated in injured cardiac microvascular endothelium, promoting neovascularization via enhanced endothelial proliferation and angiogenesis [23]. Our previous studies have also demonstrated that platelet membrane‐fused hEV accumulated in vascular endothelial cells in ischemic heart disease via the vWF/CD42b axis, thus alleviating I/RI by inhibiting cardiomyocyte apoptosis, promoting angiogenesis and increasing the number of M2 macrophages [20]. Stem cell‐derived EVs fused with leukocyte membranes exhibited enhanced cardiac targeting efficacy via Mac1/LFA1‐ICAM‐1 interaction, increasing EV accumulation and improving I/RI treatment efficacy [24]. In this study, we developed a novel bone‐marrow‐derived monocyte‐modified hEV delivery system to target the MI heart. The biomimetic system retained the biological activity of the mononuclear cell membrane and hEV with nano level, possessed good stability and biocompatibility, and could be utilized for intravenous drug administration, which was convenient for practical clinical application.

MI is one of the most serious malignant cardiovascular events in acute coronary syndromes. It is often accompanied by a vigorous inflammatory response. After myocardial ischemia, these factors will further induce and recruit inflammatory cells, exerting adverse effects on the infarcted heart [25]. Research has shown that MI triggers rapid recruitment of monocytes into the myocardium within 30 min. A robust influx of monocytes occurs, with subsequent recruitment and differentiation into macrophages at the ischemic zone and border regions between 24–72 h post‐MI [26]. In this process, some chemokines, adhesion molecules and other substances mediate the homing effect of monocytes. CD11b is mainly expressed on macrophages, monocytes, and neutrophils [19]. Studies have shown that ICAM‐1 exhibits robust upregulation on endothelial cells and persists at elevated levels on myofibers within the infarcted myocardial tissue for several days [27]. ICAM‐1 on endothelial cells and cardiomyocytes specifically binds to CD11b and is up‐regulated promptly after reperfusion injury to regulate the migration and accumulation of leukocytes in the pre‐infarction area [28]. Our study further revealed that OGD/R‐induced vascular endothelial cells and cardiomyocytes exhibited an increased expression of ICAM‐1, providing binding targets for CD11b‐expressing M‐hEV and significantly enhancing cellular uptake in the aforementioned two injured cell types. After blocking CD11b, the number of M‐hEV entering cells was notably reduced. MCP‐1 expression is upregulated in infarct and border zones post‐I/RI, facilitating neutrophil and monocyte infiltration and phagocytosis of infarcted muscle fibers [29, 30]. Clinically, elevated MCP‐1 concentrations have been detected in both the serum and cardiac tissue of individuals diagnosed with unstable angina or myocardial infarction within 0–4 h [31, 32]. MCP‐1 binds CCR2, expressed on endothelial cells and myofibroblasts, promoting inflammatory cell infiltration [33, 34]. MCP‐1 expression was upregulated in both vascular endothelial cells and cardiomyocytes following OGD/R, enhancing CCR2‐expressing M‐hEV uptake; CCR2 blockade reduced M‐hEV uptake. Post‐MI hearts exhibited monocyte recruitment via ICAM‐1 and MCP‐1 upregulation. Bone marrow‐derived mononuclear cell membranes expressing CCR2 and CD11b may home to infarcted hearts via MCP‐1/CCR2 and ICAM‐1/CD11b axes, enabling heart targeting. This homing effect was a potential target for cardio‐targeted delivery systems. This study broadens the scope of mononuclear cell membrane delivery systems in ischemic heart disease, revealing a dual targeting mechanism for injured vascular endothelial cells and cardiomyocytes. This enhances cardiac targeting and offers novel insights to overcome challenges in heart‐targeted therapies. Furthermore, previous studies have found that EVs entered cells primarily through membrane fusion and endocytosis [35]. Endocytosis involves a variety of molecular mechanisms. Clathrin‐dependent endocytosis is one of the primary molecular mechanisms for the internalization of EVs [36]. Recent studies have demonstrated that EVs engineered via cell membrane fusion, such as those cloaked with platelet or M2 macrophage membranes, exhibited distinct internalization mechanisms compared to conventional EVs [37, 38]. Rather than relying predominantly on clathrin‐mediated endocytosis, these hybrid EVs were preferentially internalized by cardiomyocytes via macropinocytosis,. Based on previous studied, it supposed that M‐hEV might further enhance the internalization of hEV within cells through macropinocytosis.

EVs have garnered growing recognition as dual‐purpose tools, serving both as diagnostic biomarkers and as candidate therapeutic agents across multiple disease contexts [39]. Some studies have identified stem‐cell‐derived EVs that contain specific miRNAs contributing to heart repair. For instance, EVs derived from MSC containing miR‐21‐5p, demonstrated significant cardioprotective effects through inhibition of the Ca^2^^+^/calmodulin‐dependent protein kinase II, effectively reducing cardiomyocyte apoptosis [40]. Experimental evidence revealed that EVs from umbilical cord‐derived stem cells containing miR‐23a‐3p conferred protection against hypoxic injury in cardiomyocytes by downregulating divalent metal transporter 1 expression, a mechanism associated with ferroptosis inhibition and myocardial damage reduction [41]. Adipose‐derived stem cell derived EVs activated the NEAT1/miR‐142‐3p/FOXO1 signaling axis, thereby providing robust anti‐apoptotic protection in cardiomyocyte [42]. However, the clinical application of EVs derived from stem cells is severely restricted by the requirements of large‐scale in vitro culture, a complex extraction process, low EV content and yield, and significant batch variations. Our previous studies have shown that hEV could repair damaged hearts. It has been found that angiogenesis was helpful in improving MI [43]. Its functional mechanism was mechanistically linked to miRNA‐486 [9]. It has been found that the upregulated miRNA‐486 expression by hypoxia‐induced mesenchymal stem cells promoted angiogenesis after MI animals, thereby enhancing cardiac function in infarcted hearts [44]. MiR‐486 exerted cardioprotective effects against cardiac I/RI injury by promoting activation of the AKT/mTOR axis [45]. EVs containing miR‐486‐5p alleviated cartilage degradation and modulates PI3K‐Akt signaling, promoting M2 macrophage polarization [46]. Moreover, previous studies have demonstrated that specific miRNAs, including miR‐24 [47], miR‐21‐5p [48], miR‐142‐3p [49], miR‐342‐5p [50], and miR‐146a [51], enriched in EVs derived from the blood under pathophysiological conditions exerted anti‐apoptotic effects in cardiomyocytes and promote pro‐reparative macrophage polarization. Our analysis revealed a statistically significant upregulation of miR‐21‐5p in both hEV and M‐hEV relative to control MSC‐EVs, indicating that miR‐21‐5p was consistently enriched in hEV and its membrane‐fused derivative. The elevated miR‐21‐5p levels contributed, at least in part, to their anti‐apoptotic effects on cardiomyocytes and vascular endothelial cells. HEV showed promise for ischemic heart disease treatment, warranting mechanistic exploration. This study revealed hEV's multi‐target effects, including anti‐apoptosis, pro‐angiogenesis, and macrophage polarization modulation. Unlike cell‐line derived EVs, circulating hEV could be obtained from healthy donors or blood banks. Using low‐speed centrifugation and molecular exclusion chromatography, a large number of hEV could be isolated from 1 mL plasma, enabling large‐scale collection. HEV treatment for ischemic heart disease is effective, with a simple, cost‐effective, and clinically feasible preparation process. Further exploration of hEV mechanisms is needed to unlock therapeutic potential. Additionally, hEV's natural origin and low immunogenicity make it an attractive candidate for clinical translation. The presence of various bioactive molecules, including miRNAs, proteins, and lipids, within hEV contributes to its therapeutic effects. Elucidating the specific molecular mechanisms underlying hEV's cardioprotective actions may identify novel targets for repair of damaged heart. Additional investigations are required to optimize hEV isolation and characterization protocols, ensuring consistency and scalability for clinical applications.

## 4 Conclusion

In conclusion, M‐hEV targeted injured vascular endothelial cells and cardiomyocytes via MCP‐1/CCR2 and ICAM‐1/CD11b axes, enhancing cardioprotection by inhibiting apoptosis, improving the inflammatory microenvironment, and promoting neovascularization, thereby augmenting cardiac repair post‐MI.

## 5 Experimental Section

### 5.1 Human Plasma and Experimental Animals

Plasma was obtained from four healthy male volunteers (aged 23–29 years). All participants provided written consent before their plasma samples were collected, in accordance with ethical guidelines. Eight‐week‐old male C57BL/6 mice were obtained from Weitong Lihua Experimental Animal Technology Co., Ltd. (Zhejiang, China). This study complied with the ethical principles of the Declaration of Helsinki and all clinical samples and animal studies were approved by the Shanghai University Committee for Animal Experiment Ethics and conducted in accordance with NIH guidelines (Approval Nos. ECSHU2024–112).

### 5.2 Isolation of hEV

HEV were extracted by using size‐exclusion chromatography with an IZON qEV column (IZON Science, New Zealand) as described in the previous study [9]. Subsequently, the eluted hEV fractions were concentrated using 100‐kDa molecular weight cutoff ultrafiltration centrifugal devices (4000 × g, 45 min).

### 5.3 Isolation of Bone‐Derived Monocytes and Preparation of Mon Nanovesicles

Mouse bone marrow‐derived monocytes were isolated by sacrificing mice, disinfecting carcasses in 75% ethanol, and aseptically harvesting femurs and tibias. The monocytes were cultured in dulbecco's modified eagle medium (DMEM) for 5 days further use [17]. The purity of monocytes was detected by flow cytometry through labeling anti‐CD11b (BD Biosciences, #563015) and anti‐mouse Ly6C (BD Biosciences, #562727). Mon nanovesicles were generated by subjecting the harvested cells to three consecutive freeze‐thaw cycles. Lastly, the samples were reconstituted in ultrapure water and subjected to bath sonication using a Fisher Scientific FS30D device (Sonics & Materials, Newtown, CT, USA) at a frequency of 20 kHz and an amplitude setting of 30% according to previous studies with minor modifications [52].

### 5.4 Preparation and Characterization of M‐hEV

Extrusion of polycarbonate membrane with a gradient pore size was employed to prepare M‐hEV. In brief, Mon nanovesicles and hEV were mixed at a 1:1 protein ratio. Membrane fusion was achieved by subjecting the mixture to sequential extrusion through polycarbonate membranes using a co‐extruder system (Avanti Polar Lipids, Inc., USA). As a control, Mon nanovesicles and hEV were simply co‐incubated without undergoing extrusion. The size distribution and Zeta potential were analyzed by NTA (Particle Metrix, Inning am Ammersee, Germany) and Zetasizer Nano ZSE (UK), respectively, and their morphology was examined by TEM (LVEM5, Quantum Design, US). To evaluate their turbidity, hEV and M‐hEV were dispersed in PBS supplemented with 10% FBS for time points ranging from 0.5 to 72 h. Absorbance readings were obtained at a wavelength of 590 nm with a SpectraMax iD3 microplate spectrophotometer (Molecular Devices, China).

### 5.5 Membrane Fusion Verification

DiD‐labeled Mon nanovesicles (Beyotime, C1039; 10 µg/mL) and DiO‐labeled hEV (Beyotime, C1038; 1 mg/mL) were first prepared by co‐incubation for 10 min. Excess dye was removed through centrifugation. M‐hEV were generated by fusing DiD‐labeled Mon nanovesicles with DiO‐labeled hEV. Confocal laser scanning microscopy (Carl Zeiss, Germany) was employed to visualize M‐hEV fusion. To confirm membrane fusion, WB was performed to detect characteristic protein markers uniquely associated with Mon nanovesicles and hEV, following established protocols from prior work [20]. The following primary antibodies were used: CD63 (ABclonal, A5271), CD9 (ABclonal, A19027), TSG101 (ABclonal, A1692), Alix (Santa Cruz, sc‐53540), Calnexin (Cell Signaling Technology, 2679), CD11b (Abclonal, A23254), PSGL‐1 (Abclonal, A23373), CCR2, Ly6C (Abclonal, A23885), and CD47 (Abclonal, A1838S). Coomassie brilliant blue staining was employed to profile the protein content of Mon nanovesicles, hEV, and M‐hEV. Protein bands were visualized using a ChemiDoc Touch Imaging System (Bio‐Rad, USA). Total protein and total RNA levels in hEV and M‐hEV were quantified using the BCA protein assay kit and RT‐qPCR, respectively. To identify the specific protein markers of M‐hEV within hybrid extracellular vesicles, nanoscale flow cytometry was performed using a CytoFLEX nano instrument (Beckman Coulter, USA). Briefly, M‐hEV were incubated with APC/Cyanine7 anti‐mouse CCR2 (Biolegend, 150642) and FITC anti‐human CD63 (Biolegend, 353006) followed by removal of unbound antibodies. Double‐positive (CD63^+^/CCR2^+^) M‐hEV were subsequently identified and analyzed by the nano flow cytometer. TEM was employed to analyze the membrane orientation of fusion‐derived hybrid vesicles. Briefly, M‐hEV were initially incubated with an anti‐CCR2 antibody and anti‐CD11b antibody, followed by incubation with a secondary anti‐rabbit IgG conjugate (Abcam, ab41498). The samples were rinsed sequentially with deionized water to remove unbound reagents, followed by TEM imaging.

### 5.6 Isolation and Culture of NRCMs

The isolation and culture of NRCMs were performed following an established protocol [9]. Briefly, cardiac tissue was aseptically harvested from newborn SD rats (1‐3 days postnatal) and subsequently fragmented into approximately 1 mm^2^ sections under cold conditions. Tissue digestion was achieved through enzymatic treatment using a combination of Collagenase II (Gibco, catalog number 17101015) and porcine pancreatic trypsin (Sigma, catalog number P3292). Following isolation, the NRCMs were cultured in DMEM supplemented with 5% FBS and 10% equine serum for subsequent experimental applications.

### 5.7 OGD/R Model

OGD/R injury model was established by exposing HUVECs and NRCMs to glucose‐ and serum‐free DMEM under hypoxic conditions for 8 h. Then, both cell types were returned to standard complete DMEM containing glucose and serum and cultured under normoxic conditions for an additional 12 h. This OGD/R protocol was employed to induce cell death in HUVECs and NRCMs.

### 5.8 HUVECs and NRCMs Binding Assay

Uptake of hEV and M‐hEV by HUVECs and NRCMs was visualized and assessed using laser scanning confocal microscopy. OGD/R‐injured HUVECs and NRCMs were seeded into an 8‐well µ‐Slide (Ibidi GmbH, Germany) at densities of 1 × 10^4^ and 2 × 10^4^ cells per well, respectively. Subsequently, PBS, DiD‐labeled hEV, or DiD‐labeled M‐hEV were administered to the above two cell lines at a concentration of 1 × 10^10^ particles/mL. For blocking assays, HUVECs and NRCMs were incubated with anti‐ICAM‐1 (sc‐8439, Santa Cruz) and anti‐MCP‐1 (sc‐52701, Santa Cruz) antibodies. HEV or M‐hEV were pre‐incubated with or without anti‐CD11b antibody (Abclonal, A23254) (4.54 mg/mL), anti‐CCR2 antibody (Abclonal, A2855) (1.38 mg/mL) for blocking in medium for 0.5 h prior to cellular exposure. Following incubation, the unbound antibodies were removed by three consecutive washes with PBS before use [24]. The cells were then stained with rabbit anti‐vWF antibody (Proteintech, 27186‐1‐AP) in HUVECs and anti‐α‐actinin antibody in NRCMs (Sigma–Aldrich, A7811) and nuclei were counterstained with Hoechst33342 (KeyGEN BioTECH, KGA212‐1). Fluorescence intensity in HUVECs and NRCMs following treatment with the indicated experimental groups was quantified by flow cytometry (Beckman Coulter, Suzhou, China).

### 5.9 TUNEL Staining In Vitro

HUVECs and NRCMs subjected to OGD/R were seeded in 12‐well plates at densities of 1 × 10^5^ and 4.2 × 10^5^ cells/mL, respectively. After stabilization, cells were exposed for 12 h to PBS, hEV, or M‐hEV (1 × 10^10^ particles/mL). The HUVECs and NRCMs were then fixed with 4% paraformaldehyde, and cell apoptosis was quantified using the TUNEL FITC Apoptosis Detection Kit (Vazyme, A111‐03), following the manufacturer's instructions.

### 5.10 WB Analysis

WB was performed to quantify the expression levels of critical regulatory proteins involved in apoptotic signaling. Briefly, cells subjected to the previously described in Section 5.9 were harvested and lysed on ice in RIPA lysis buffer (Beyotime, P0013B) supplemented with 1% phenylmethylsulfonyl fluoride as well as a cocktail of protease and phosphatase inhibitors (Beyotime, ST2573). Following lysis, the samples were subjected to centrifugation at 12,000 × g for 20 min at 4 °C. Equivalent quantities of total protein (10–30 µg per lane) were electrophoretically separated using SDS‐polyacrylamide gel electrophoresis and then blotted onto polyvinylidene fluoride membranes. Immunodetection was carried out using the following primary antibodies: anti‐Bax (Abclonal, A19684), anti‐Bcl2 (Affinity Biosciences, AF6138), and anti‐caspase3 (Abclonal, A2156). 𝛽‐Actin (Abclonal, AC004) was used as a loading control.

### 5.11 Endothelial Cell Migration and Lumen Formation

Cell migration was firstly assessed using a transwell system. OGD/R‐treated HUVECs (1 × 10^5^ cells/mL) were plated into the upper compartment and exposed to PBS, hEV, or M‐hEV (1 × 10^10^ particles/mL). Following a 12‐h incubation period, the cells were fixed using 4% paraformaldehyde and subsequently stained with 0.5% crystal violet (Beyotime, C0121). In the wound healing assay, HUVECs were plated in 12‐well culture plates at a seeding density of 1 × 10^5^ cells/well and subjected to OGD/R stress. A standardized linear wound was generated across the confluent HUVEC monolayer using a sterile pipette tip under aseptic conditions, ensuring perpendicular orientation to the well's central axis. Initial microscopic images were captured at baseline (0 h), followed by treatment administration with PBS, hEV, and M‐hEV (1 × 10^10^ particles/mL). After 12 h of incubation, secondary images were acquired, and wound closure was quantified using ImageJ by measuring the relative wound area, defined as the ratio of wound area at 12 h to that at 0 h. The capacity of cells to induce angiogenesis was evaluated using a tube formation assay conducted on Matrigel‐coated surfaces. OGD/R‐stressed HUVECs (1.5 × 10^5^ cells/mL) were seeded onto Ceturegel Matrigel (40183ES08, Yeasen)‐coated 96‐well plates and treated with PBS, hEV, or M‐hEV (1 × 10^10^ particles/mL). After 12 h of treatment, capillary‐like structure formation was examined microscopically, with branch point quantification performed using ImageJ software. No OGD/R‐induced HUVECs served as experimental controls in all above assays.

### 5.12 Macrophage Polarization In Vitro

To induce macrophage polarization, RAW264.7 (10^5^ cells/well) of were co‐incubated with 100 ng/mL LPS (Sigma, L4391) to promote M1 phenotype or 20 ng/mLIL‐4 (Sino Biological, 51084‐MNAE) to drive M2 polarization. Following PBS, hEV, or M‐hEV (1 × 10^10^ particles/mL) treatments for 24 h, macrophage phenotype characterization was performed by flow cytometry. M1 macrophages were identified through staining with PE‐Cyanine7‐conjugated anti‐CD86 (B7‐2) monoclonal antibody (clone GL1; eBioscience, 25‐0862‐82), while M2 macrophages were detected using APC‐eFluor 780‐labeled anti‐CD206 (MMR) monoclonal antibody (clone MR6F3; Invitrogen, 17‐2061‐80).

### 5.13 MI Model and In Vivo Targeting Assay

MI was surgically induced in mice via permanent ligation of the left anterior descending coronary artery (LAD). No LAD ligation mice were used as control, and all animals were humanely euthanized 24 h after the experimental intervention. For in vivo targeting, mice were administered intravenously with PBS, DiD‐labeled hEV, or DiD‐labeled M‐hEV (10 µg/mL DiD, 2.5 × 10^10^ particles) 10 min post‐MI. For blocking assays, M‐hEV were pre‐incubated with anti‐CD11b (4.54 mg/mL) or anti‐CCR2 (1.38 mg/mL) for 0.5 h. Following incubation, the unbound antibodies were removed by three consecutive washes with PBS before use [24]. For targeting assay i*n vivo*, mice with acute MI were sacrificed at 24 h post‐infarction. Hearts and major organs were imaged using a in vivo imaging system (PerkinElmer, USA). To assess M‐hEV distribution in infarcted myocardium, immunofluorescence staining was performed using an anti‐α‐actinin antibody to label cardiomyocytes in the injured regions of the heart.

### 5.14 TTC Staining

To investigate the therapeutic effect of M‐hEV on acute MI mice in vivo, animals were administrated PBS, hEV, and M‐hEV (2.5 × 10^10^ particles/mL) via intravenous injections after 10 min of MI surgery. Hearts were harvested 24 h post‐MI, sectioned into 1‐mm‐thick slices, and subjected to TTC staining after Evans blue injection. The AAR/LV ratio was calculated to confirm consistency of MI induction across animals. The INF/AAR ratio was utilized to assess myocardial infarction size.

### 5.15 Myocardial Apoptosis In Vivo

On day 1 post‐treatment, 10‐µm cryosections of infarcted mouse hearts were fixed with 4% paraformaldehyde and subjected to TUNEL staining (Promega, G3250). Myocardial cells were visualized via immunofluorescent labeling of α‐actinin. Nuclear counterstaining was performed with Hoechst 33342, and confocal images were captured with a Zeiss LSM 710. The expressions of proteins in heart tissues were analyzed by WB in accordance with section 5.10.

### 5.16 Immunohistochemistry and Immunofluorescence

At treatment day 3, hearts from MI mice were harvested, cryosectioned (6 µm), and subjected to CD31 immunohistochemical staining (Cell Signaling Technology, 77699S). The biological microscope (Leica, DM3000) was employed to capture images related to angiogenesis. Subsequently, Blood vessel count and density were quantified using ImageJ software. Three days post‐treatment, immunohistochemical staining was used to investigate macrophage polarization status in hearts of MI mice using specific markers. The M1 macrophage subpopulation was labeled by anti‐iNOS antibody (Thermo Fisher Scientific, PA1‐036), while M2 macrophages were labeled by anti‐CD206 antibody (Thermo Fisher Scientific, MA5‐16871).

### 5.17 Echocardiography and Masson Staining

MI‐induced mice received weekly intravenous injections of PBS, hEVs, or M‐hEVs (2.5 × 10^10^ particles) for three consecutive weeks. Cardiac function, assessed via echocardiography using the Vevo2100 system (FUJIFILM Visual Sonics), was evaluated for EF and FS at the end of treatment. To quantify myocardial fibrosis, Paraffin‐embedded cardiac tissue samples were sectioned at a thickness of 5 µm and stained with Masson's trichrome dye (Solarbio, G1340) following the manufacturer's instructions.

### 5.18 RT‐qPCR Assay

Total RNA was extracted from murine cardiac tissues with TRIzol RNAiso Plus (TaKaRa) and subsequently converted into complementary DNA (cDNA) using RevertAid First Strand cDNA Synthesis Kit (Thermo Fisher Scientific, K1622). Total RNA from MSC‐EVs, hEV, and M‐hEV was isolated by miRNeasy Mini Kit (217004, QIAGEN, Germany). RNA was reverse transcribed to complementary DNA (cDNA) using the RevertAid First Strand cDNA Synthesis Kit (K1622, Thermo Fisher Scientific, USA). The mRNA and miRNA levels were analyzed by RT‐qPCR using ChamQ Universal SYBR qPCR Master Mix (Vazyme, Q711‐03) on a Roche LightCycler480 PCR System. 18S was served as the endogenous reference gene. The corresponding primer sequences were summarized in Table S1 for inflammatory factors and Table S2 for pathological markers of the heart. The reverse transcription primers and the RT‐qPCR primers of miRNA and U6 (internal control) were purchased from RiboBio, China.

### 5.19 Biocompatibility of M‐hEV

The cytotoxicity of M‐hEV was assessed in HUVECs and NRCMs cultured in 96‐well microplates (1 × 10^4^ and 2 × 10^4^ cells per well). Experimental groups received PBS, hEV, or M‐hEV (1 × 10^9^ particles/mL), followed by 48 h incubation under standard culture conditions. Then, the cytotoxicity of the above two types of cells was evaluated using a CCK‐8 kit (Beyotime, C0037) according to the manufacturer's protocol. For in vivo biocompatibility evaluation, PBS, hEV, or M‐hEV (2.5 × 10^10^ particles) were injected intravenously in mice. Post‐administration (24 h), major organ specimens including brains, livers, spleens, lungs, and kidneys were harvested for histopathological analysis. Tissue sections underwent standard H&E staining protocols to evaluate potential morphological alterations.

### 5.20 Statistical Analysis

In the experiments described above, each group or condition included at least three independent biological replicates. Relevant data were presented as mean ± standard deviation and analyzed using GraphPad Prism software (version 8.0, USA). Sample sizes (n) were indicated in each figure legend. For comparisons of two or multiple groups, distribution of data was first analyzed by Shapiro‐Wilk test or Kolmogorov‐Smirnov test. For normally distributed variables, an unpaired Student's t‐test was performed for comparisons between two groups; one‐way ANOVA or two‐way ANOVA followed by Bonferroni post hoc tests were performed for comparisons for multiple groups. For non‐normally distributed variables, statistical analyses were performed using the Mann‐Whitney U test for two‐group comparisons and the Kruskal‐Wallis test followed by Benjamini‐Hochberg corrected post hoc multiple comparisons for three or more groups. A *p* value < 0.05 was considered statistically significant. Data were normalized to the control group where necessary to minimize inter‐experimental variation.

## Funding

This work was supported by the grants from National Natural Science Foundation of China (82200321 to Q. L. Z), Shanghai Sailing Program (21YF1413200 to Q. L. Z), Science and Technology Commission of Shanghai Municipality (22ZR1423100 to J. Z. J).

## Conflicts of Interest

The authors declare that they have no conflicts of interest.

## Supporting information




**Supporting File**: advs75445‐sup‐0001‐SuppMat.docx

## Data Availability

The data that support the findings of this study are available from the corresponding author upon reasonable request.
