# Supplementary material for: Biomimetic Bone Marrow Monocyte Membrane‐Fused Extracellular Vesicles for Targeted Therapy of Myocardial Infarction
Source: Adv Sci (Weinh). 2026 Apr 27;13(41):e75445. doi: 10.1002/advs.75445 (PMC13335711; doi:10.1002/advs.75445)
Supplement: Supplementary file 1 — Supporting File: advs75445‐sup‐0001‐SuppMat.docx [file ADVS-13-e75445-s001.docx]

**Biomimetic bone marrow monocyte** **membrane-fused extracellular vesicles for targeted therapy of myocardial infarction**

***Jiaxin Song^1,2,#^, Hao Yang^1,2,#^, Qiqi Zhang^1,2^, Wenqin Zhou^1,2^, Rui Wang^1,2^, Yuxing Xie^1,2^, Emeli Chatterjee^3^, Guoping Li^4^, Jizong Jiang******^1,2, *^, Qiulian Zhou^1,2,*^, Cuimei Zhao^5*^***

^1^Institute of Geriatrics (Shanghai University), Affiliated Nantong Hospital of Shanghai University (The Sixth People's Hospital of Nantong) and School of Life Science, Shanghai University, Nantong 226011, China

^2^Cardiac Regeneration and Ageing Lab, Institute of Cardiovascular Sciences, Shanghai Engineering Research Center of Organ Repair, Joint International Research Laboratory of Biomaterials and Biotechnology in Organ Repair (Ministry of Education), School of Life Science, Shanghai University, Shanghai 200444, China

^3^Cardiovascular Division of the Massachusetts General Hospital and Harvard Medical School, Boston, MA 02114, USA

^4^Department of Anesthesia, Critical Care and Pain Medicine, Massachusetts General Hospital and Harvard Medical School, Boston, MA 02114, USA

**^5^Department of Cardiology, Shanghai Tongji Hospital, Tongji University School of Medicine, Shanghai 200065, China**

*Correspondence should be addressed to

Dr. Jizong Jiang Email: jiangjizong@shu.edu.cn

Dr. Qiulian Zhou E-mail: [zhouqiulian@shu.edu.cn](mailto:zhouqiulian@shu.edu.cn) or qlzhou_shu@163.com

Dr. Cuimei Zhao E-mail: [zhaocuimei2000@126.com](mailto:zhaocuimei2000@126.com)


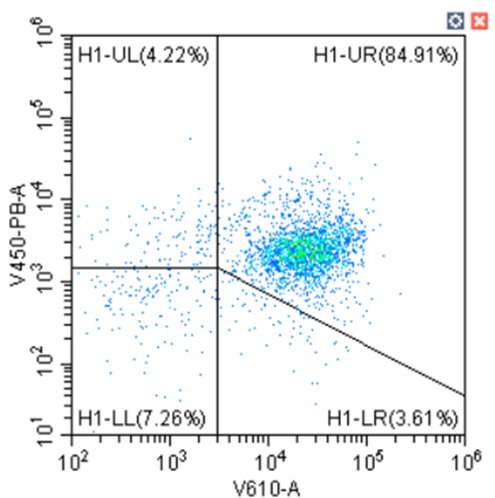


Figure S1 Flow cytometry was employed to investigate the phenotyping of bone marrow cell suspensions. Bone marrow monocytes were isolated and subsequently labeled with the fluorescent antibodies of anti-Ly6C and anti-CCR2. The percentage of Ly6C^+^/CCR2^+^ were further analyzed by flow cytometry. Flow cytometry data are representative for three individual experiments (n = 3).


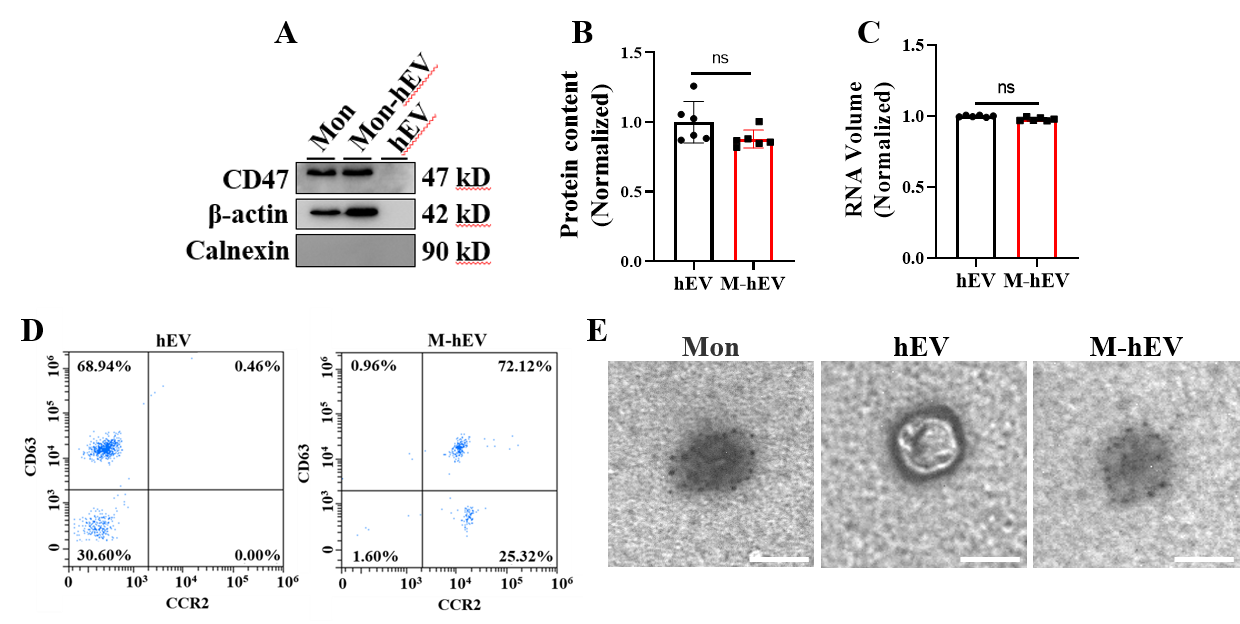


Figure S2 Verification of membrane fusion of M-hEV. (A) Expression of CD47 on Mon, hEV, and M-hEV (n = 3). (B) BCA kit analysis for the total protein contents of hEV and M-hEV (n = 6). (C) Rt-qPCR analysis was conducted for the total RNAs of hEV and M-hEV (n = 6). (D) Nano-flow cytometry for quantifying the portion of CD63⁺/CCR2⁺ M-hEV within the hybrid vesicles (n = 3). (E) Immunogold TEM of M-hEV using anti-CD11b and anti-CCR2 primary antibodies, followed by colloidal gold-conjugated goat anti-rabbit IgG (n = 3) (Scale bar=100 nm). The data were presented as the mean ± standard deviation. An unpaired Student’s t-test was performed (B and C), ns, not significant.

Figure S3 RT-qPCR analysis of miR-24 (A) (n = 6), miR-21-5p (B) (n = 6), miR-142-3p (C) (n = 6), miR-342-5p (D) (n = 6), and miR-146a (E) (n = 6) expression levels in MSC-EV, hEV, and M-hEV. The data were presented as the mean ± standard deviation. One-way ANOVA with Bonferroni correction (B, C and E), non-parametric Kruskal-Wallis test with the Benjamini-Hochberg correction (A and D) were performed, *** *p* < 0.001.


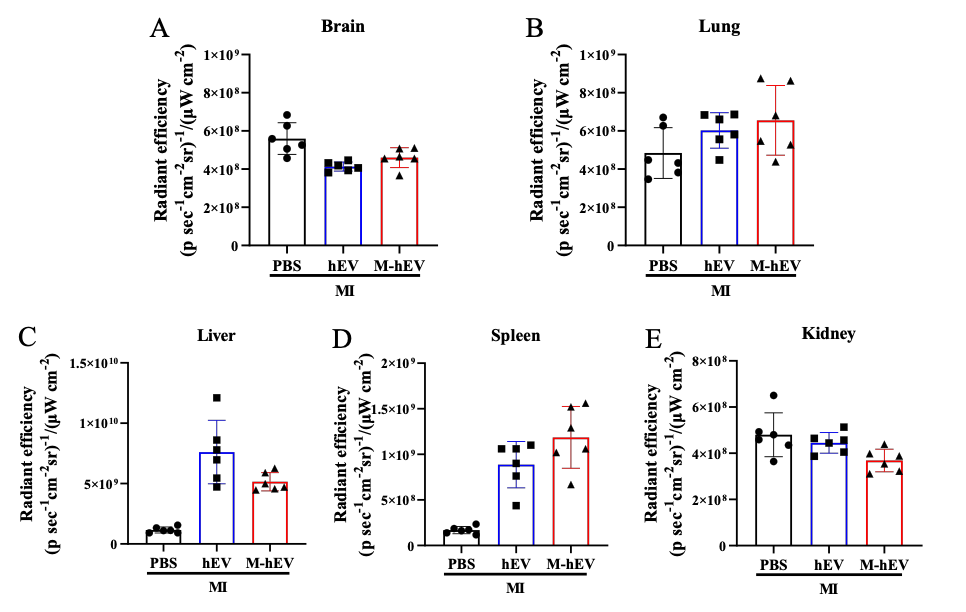


Figure S4 The distribution of M-hEV in the main organs of MI mice. After the DiD-labeled hEV and M-hEV being intravenously injected into mice, the fluorescence intensity in each organ was analyzed by IVIS spectrum. (A) Brain (n = 6). (B) Lung (n = 6). (C) Liver (n = 6). (D) Spleen (n = 6). (E) Kidney (n = 6). The data were presented as the mean ± standard deviation. One-way ANOVA with Bonferroni correction was performed (A-E).

Figure S5 The *in vivo* targeting of M-hEV after pre-incubation with specific antibodies (anti-CCR2 and anti-CD11b) for blocking purposes. 24 h after intravenous administration of DiD-labeled hEV, DiD-labeled M-hEV, and DiD-labeled M-hEV pre-incubated with anti-CCR2 or anti-CD11b antibody, ex vivo fluorescence imaging was performed on major harvested organs. (A) *Ex vivo* fluorescence imaging of hearts (n = 3). (B) *Ex vivo* fluorescence imaging of main organs, including brains, livers, spleens, kidneys, and lungs (n = 3).

Figure S6 The biocompatibility of M-hEV. (A&B) After 48 h incubation, cell viabilities of HUVECs (A) (n = 6) and NRCMs (B) (n = 6) were evaluated by CCK8 kit. (C) PBS, hEV, and M-hEV were intravenously injected into mice. After 24 h, the brains, livers, spleens, lungs, and kidneys of the mice were harvested for H&E staining to investigate the histological pattern of each organ (n = 3) (Scale bar = 25 μm). The data were presented as the mean ± standard deviation. One-way ANOVA with Bonferroni correction was performed (A and B).

、

Table S1 Primer sequences for qRT-PCR analysis of inflammatory factors

| Gene | Primer (5’-3’) | |
| --- | --- | --- |
| 18S-F | TCAAGAACGAAAGTCGGAGG |  |
| 18S-R | GGACATCTAAGGGCATCAC |  |
| mmu-IL-1β-F | GAAGAGCCCATCCTCTGTGA |  |
| mmu-IL-1β-R | GGGTGTGCCGTCTTTCATTA |  |
| mmu-IL-6-F | GAGGATACCACTCCCAACAGACC |  |
| mmu-IL-6-R | AAGTGCATCATCGTTGTTCATAC |  |
| mmu-TNF-α-F | CGTCAGCCGATTTGCTATCT |  |
| mmu-TNF-α-R | CGGACTCCGCAAAGTCTAAG |  |
| mmu-IL-10-F | GGCCCAGAAATCAAGGAGCA |  |
| mmu-IL-10-R | GCCTTGTAGACACCTTGGTCTT |  |

Table S2 Primer sequences for qRT-PCR analysis of cardiac pathological biomarkers

| Gene | Forward primer (5′-3′) | Reverse primer (5′-3′) |
| --- | --- | --- |
| mmu-ANP | GAGCAAATCCCGTATACAGTGC | ATCTTCTACCGGCATCTTCTCC |
| mmu-BNP | CTGCTTGCGGAGGCGAGAC | TGTTCTGGAGACTGGCTAGGACTTC |
| mmu-β-MHC | CAGAACACCAGCCTCATCAACCAG | TTCTCCTCTGCGTTCCTACACTCC |
| mmu-Collagen I | CCCTGAAGTCAGCTGCATACACAA | CCTACATCTTCTGAGTTTGGTGAT |
| mmu-Collagen III | GAGATGTCTGGAAGCCAGAACCAT | GATCTCCCTTGGGGCCTTGAGGT |
| 18 s | TCAAGAACGAAAGTCGGAGG | GGACATCTAAGGGCATCAC |
